# A novel four‐gene signature predicts immunotherapy response of patients with different cancers

**DOI:** 10.1002/jcla.24494

**Published:** 2022-05-19

**Authors:** Yuanli Liu, Mingyue Ni, Lamei Li, Junyan Wang, Zhenzhen Tu, Haisheng Zhou, Siping Zhang

**Affiliations:** ^1^ Department of Biochemistry and Molecular Biology, School of Basic Medical Sciences Anhui Medical University Hefei China; ^2^ Department of Dermatology Anhui Provincial Hospital Affiliated to Anhui Medical University Hefei China; ^3^ Department of Clinical Medicine (5+3 Programme) Anhui Medical University Hefei China

**Keywords:** cancer, *CD8*, immune checkpoint‐blockade, *LEF1*, *TCF7*

## Abstract

**Background:**

Immune checkpoint blockade (ICB) therapy has demonstrated favorable clinical efficacy, particularly for advanced or difficult‐to‐treat cancer types. However, this therapy is ineffective for many patients displaying lack of immune response or resistance to ICB. This study aimed to establish a novel four‐gene signature (*CD8A*, *CD8B*, *TCF7*, and *LEF1*) to provide a prognostic immunotherapy biomarker for different cancers.

**Methods:**

Transcriptome profiles and clinical data were obtained from The Cancer Genome Atlas database. Multivariate Cox regression analysis was used to establish a four‐gene signature. The R package estimate was used to obtain the immune score for every patient.

**Results:**

Risk scores of the novel four‐gene signature could effectively divided all patients into high‐ and low‐risk groups, with distinct outcomes. The immune score calculated via the estimate package demonstrated that the four‐gene signature was significantly associated with the immune infiltration level. Furthermore, the four‐gene signature could predict the response to atezolizumab immunotherapy in patients with metastatic urothelial cancer.

**Conclusions:**

The novel four‐gene signature developed in this study is a good prognostic biomarker, as it could identify many kinds of patients with cancer who are likely to respond to and benefit from immunotherapy.

## Graphical abstract

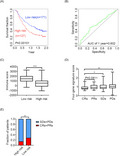

Multivariate Cox regression analysis was used to establish a novel four‐gene signature, which was significantly associated with the immune infiltration level. The four‐genesignature could predict the response to atezolizumab immunotherapy in patients withmetastatic urothelial cancer. The novel four‐gene signature developed in this study isa good prognostic biomarker, as it could identify many kinds of patients with cancerwho are likely to respond to and benefit from immunotherapy.

## 1 INTRODUCTION

Cancer immunotherapy using immune checkpoint blockade (ICB) drugs that target programmed cell death 1 (PD1), programmed cell death 1 ligand 1 (PD‐L1), and cytotoxic T‐lymphocyte‐associated protein 4 (CTLA‐4) is a promising treatment strategy that can produce a durable response, especially for patients with metastatic cancers. Nevertheless, many patients with cancer fail to respond to immunotherapy or achieve durable remission. The objective response rate of ICB is maintained at 13%–27.3% in many tumors, such as non‐small‐cell lung cancer (NSCLC),^1^, ^2^, ^3^, ^4^, ^5^ urothelial carcinoma,^6^ renal cell carcinoma (RCC),^7^, ^8^, ^9^, ^10^ triple‐negative breast cancer (TNBC),^11^ liver hepatocellular carcinoma (LIHC),^12^, ^13^ thyroid carcinoma (THCA),^14^ adrenocortical carcinoma (ACC), and uveal melanoma (UVM).^15^ A small percentage of patients receiving anti‐CTLA‐4/B7 or anti‐PD1/PD‐L1 therapy experienced prolonged survival. Thus, the combination of anti‐CTLA‐4/B7 with anti‐PD1/PD‐L1 therapy may have promising clinical efficacy.^16^ To apply immunotherapy to many types of cancers, it is pivotal to predict the immune responses and clinical outcomes of patients with metastatic cancer prior to treatment, and to enhance the response to immunotherapy of recalcitrant patients, whose cancer cells may be more resistant to ICB agents.

Previous studies have reported that PD‐L1 expression in tumor cells can be used to predict clinical responses to ICB therapy.^5^, ^17^, ^18^ However, some patients with PD‐L1‐negative melanoma can also derive durable clinical benefits from PD‐1 blockade.^19^ Notably, T cells play a crucial role in immune defense against cancer.^20^, ^21^, ^22^, ^23^ In particular, the level of tumor‐infiltrating CD8^+^ T lymphocytes is often a predictor of patient survival and response to immunotherapy,^24^, ^25^, ^26^, ^27^, ^28^, ^29^, ^30^, ^31^ and both anti‐CTLA‐4 and anti‐PD‐1 treatments can induce CD8^+^ T‐cell expansion. However, not all CD8^+^ T‐cell subsets exhibit this behavior.^32^

Some memory CD8^+^ T cells in human lymph nodes are highly similar to a subset of mouse CD8^+^ T cells, identified in chronic infection models, that respond to checkpoint blockade immunotherapy. These cells exhibit a distinct transcriptional signature, including the expression of lymphoid enhancer binding factor 1 (*LEF1*) and T‐cell‐specific transcription factor 7 (*TCF7*).^33^ TCF7 and LEF1 are historically known as effector transcription factors acting downstream of the WNT signaling pathway, and are essential for early T‐cell development.^34^, ^35^ A group of CD8^+^ T cells displaying hallmarks of exhausted cells and central memory cells was identified; notably, *TCF7* expression was required for the generation of this CD8^+^ T‐cell subset, which exhibited a proliferative burst after PD1 blockade.^34^, ^36^, ^37^ The “progenitor” or “stem‐like” exhausted cells were a subset of exhausted CD8^+^ tumor‐infiltrating lymphocytes that persisted long term and retained polyfunctionality. Moreover, melanoma patients displaying a higher percentage of progenitor exhausted cells experienced more durable response to checkpoint blockade therapy.^38^ Stem‐like CD8^+^ T cells in human tumors have been confirmed to be stem‐like CD8^+^ T cells expressing *TCF7*, which divide into terminally differentiated cells that express effector molecules. Additionally, initiation of effector differentiation is critical for the infiltration of many T cells into the tumor.^39^

*TCF7 and LEF1*, belonging to the high‐mobility group (HMG) family owing to their conserved HMG DNA‐binding domains, are well‐known stem cell‐associated transcription factors that are usually expressed in CD8^+^ T cells,^40^, ^41^ and play critical roles in establishing CD8^+^ T‐cell identity through their intrinsic histone deacetylase activity.^42^, ^43^ These proteins also play vital roles in the regulation of CD8^+^ T‐cell function and differentiation.

The CD8 antigen, acting as a co‐receptor of T lymphocytes, is composed of the isoforms CD8 alpha chain and CD8 beta chain, which are encoded by *CD8A* and *CD8B*, respectively. Granzyme A (GZMA) and granzyme B (GZMB) are T cell‐ and natural killer cell‐specific serine proteases, which may function as common components necessary for the lysis of target cells by cytotoxic T lymphocytes (CTLs) and natural killer cells. Perforin 1 (PRF1) forms membrane pores that allow the release of granzymes and the subsequent cytolysis of target cells. Previous studies have shown that the average expression levels of *CD8A*, *CD8B*, *GZMA*, *GZMB*, and *PRF1* can be used to estimate CTL levels in a tumor.^44^ Therefore, *CD8A*, *CD8B*, *TCF7*, and *LEF1* expression may reveal the presence of stem‐like CD8^+^ T cells and may be thus used as a reliable immunotherapy biomarker to predict ICB outcomes. In the current study, we established an immune‐related four‐gene signature (*CD8A*, *CD8B*, *TCF7*, and *LEF1*) based on multivariable Cox regression analysis of transcriptome profiles downloaded from The Cancer Genome Atlas (TCGA) database, and assessed whether the four‐gene signature could predict the clinical responses and treatment benefits of patients with various types of cancer before the beginning of immunotherapy.

## 2 MATERIALS AND METHODS

### 2.1 Patient cohorts

Transcriptome profiles (RNA‐seq profiles) and clinical information were obtained from the TCGA database (https://cancergenome.nih.gov/). Reads per kilobase per million (RPKM) indicates the gene expression levels in different cancer patients. Ten types of cancers were included in this study: Breast cancer (BRCA), skin cutaneous melanoma (SKCM), lower‐grade glioma (LGG), kidney renal papillary cell carcinoma (KIRP), rectum adenocarcinoma (READ), kidney renal clear cell carcinoma (KIRC), THCA, LIHC, ACC, and UVM (Table S1). Data from patients with metastatic urothelial cancer (mUC) treated with atezolizumab (an anti‐PD‐L1 agent) were obtained from the R package IMvigor210CoreBiologies (version 1.0.0).^45^

### 2.2 Statistical analysis

All statistical and bioinformatics analyses were performed in R (version 4.0.1).

### 2.3 Identification of Differentially Expressed Genes (DEGs)

Differential expression analysis for the four genes *CD8A*, *CD8B*, *TCF7*, and *LEF1* between tumor and normal tissues was performed using edge R (version 3.30.3).

### 2.4 Functional and pathway enrichment analyses

Gene Ontology (GO) and Kyoto Encyclopedia of Genes and Genomes (KEGG) analyses were performed using the R package clusterProfiler (version 3.16.0) to investigate the functional and pathway enrichment associated to the four genes *CD8A*, *CD8B*, *TCF7*, and *LEF1*. The terms were sorted by their *p* value.

### 2.5 Cox proportional hazards regression model and risk score

The four‐gene signature (*CD8A*, *CD8B*, *TCF7*, and *LEF1*) was created as a prognostic model that was used to calculate the risk score of each patient based on multivariable Cox regression analysis. The mean risk score value was used as a threshold, according to which the patients were divided into high‐ and low‐risk groups. The C‐index was used to evaluate the validation of the four‐gene signature prognostic model. Time‐dependent receiver operating characteristic (ROC) curves were used to assess the sensitivity and specificity of the four‐gene signature. Multivariate Cox regression analysis was performed using the R package survival (version 3.2–3).

### 2.6 Survival curve analysis

Survival results were expressed as Kaplan–Meier (KM) curves, and statistical significance was assessed using the log‐rank test. Receiver operating characteristic (ROC) curve analysis was performed using the R package survival ROC (version 1.0.3). Statistical significance was set at *p* < 0.05.

### 2.7 Immune infiltration analysis

Immune scores, which represent the immune infiltration levels of patients, were calculated with the R package estimate (version 1.0.13)^46^ based on the TCGA RNA‐seq database.

## 3 RESULTS

### 3.1 Prognosis prediction for BRCA

**FIGURE 1:**
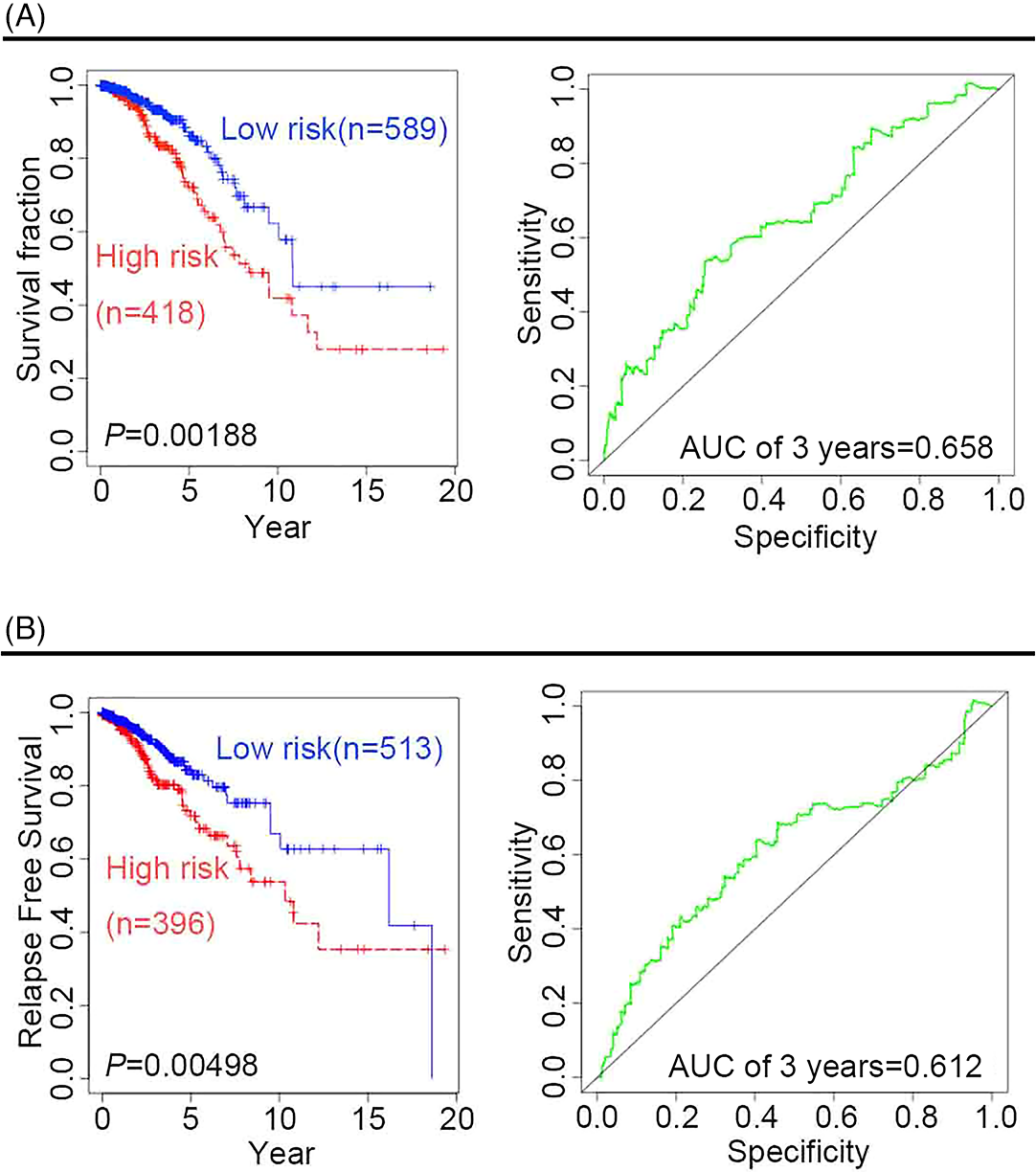
Survival analysis of patients with BRCA. (A) OS analysis of the four‐gene signature (*CD8A*, *CD8B*, *TCF7*, and *LEF1*) in BRCA and ROC curves for the 3‐year OS. (B) RFS analysis of the four‐gene signature in BRCA and ROC curves for the 3‐year RFS

To assess the predictive ability of the four‐gene signature (*CD8A*, *CD8B*, *TCF7*, and *LEF1*), multivariate analysis was performed using Cox proportional hazard regression for patients with BRCA. The resulting KM survival curves and log‐rank tests pointed at significant differences in both clinical survival outcomes (Figure 1A) and relapse‐free survival (RFS) outcomes (Figure 1B) between high‐risk and low‐risk patients (*p* < 0.01), confirming the robustness of the four‐gene signature predictive capacity. The analysis of time‐dependent ROC curves revealed that the 3‐year overall survival (OS) and RFS of the area under the curve (AUC) were 0.658 and 0.612, respectively.

**FIGURE 2:**
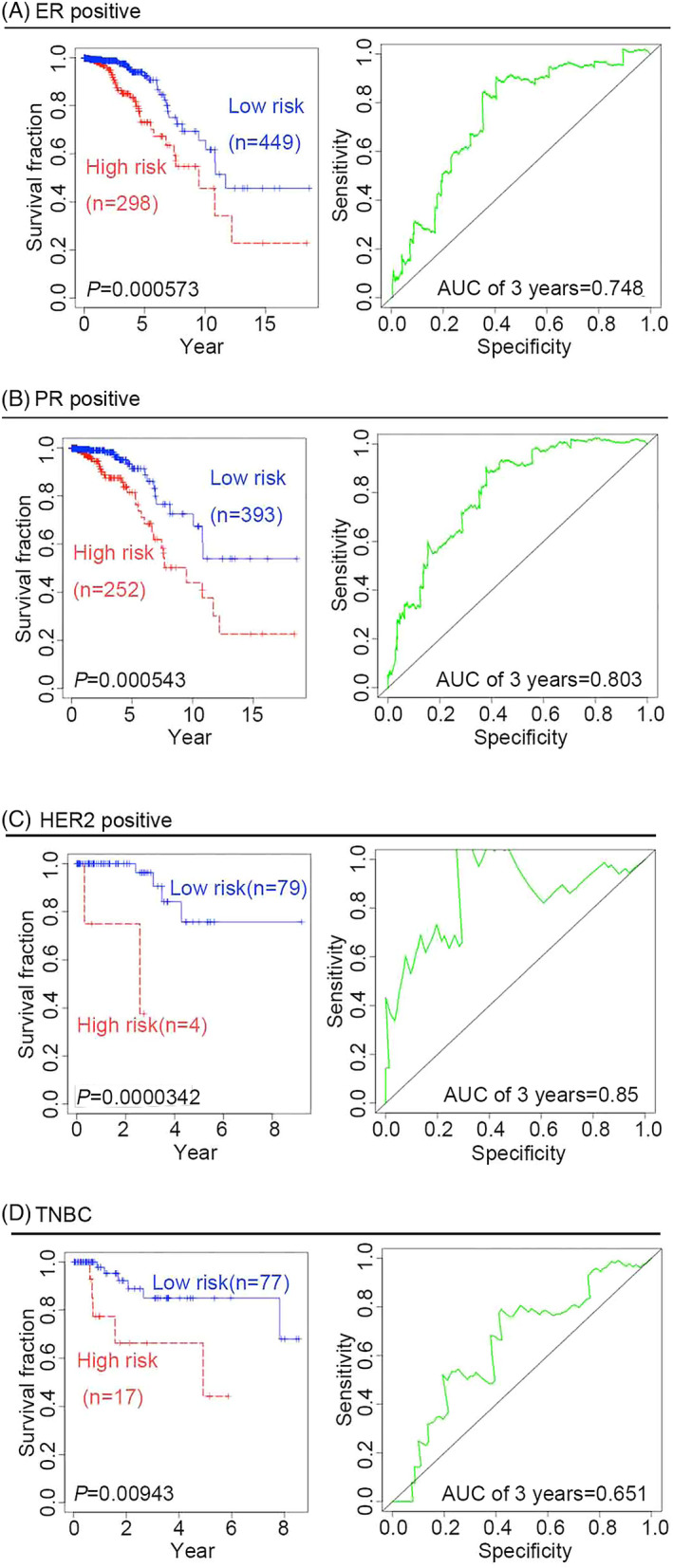
Overall survival analysis and ROC curves in different pathological subtypes of BRCA. The KM curves of the four‐gene signature (*CD8A*, *CD8B*, *TCF7*, and *LEF1*) showed that the risk score could be effectively used to divide patients into high‐ and low‐risk groups with distinct outcomes. The AUC of the ROC curve was used to evaluate the ability of the four‐gene signature to predict prognosis in terms of OS. (A) Patients with ER‐positive BRCA. (B) Patients with PR‐positive BRCA. (C) Patients with HER2‐positive BRCA. (D) Patients with TNBC BRCA

**FIGURE 3:**
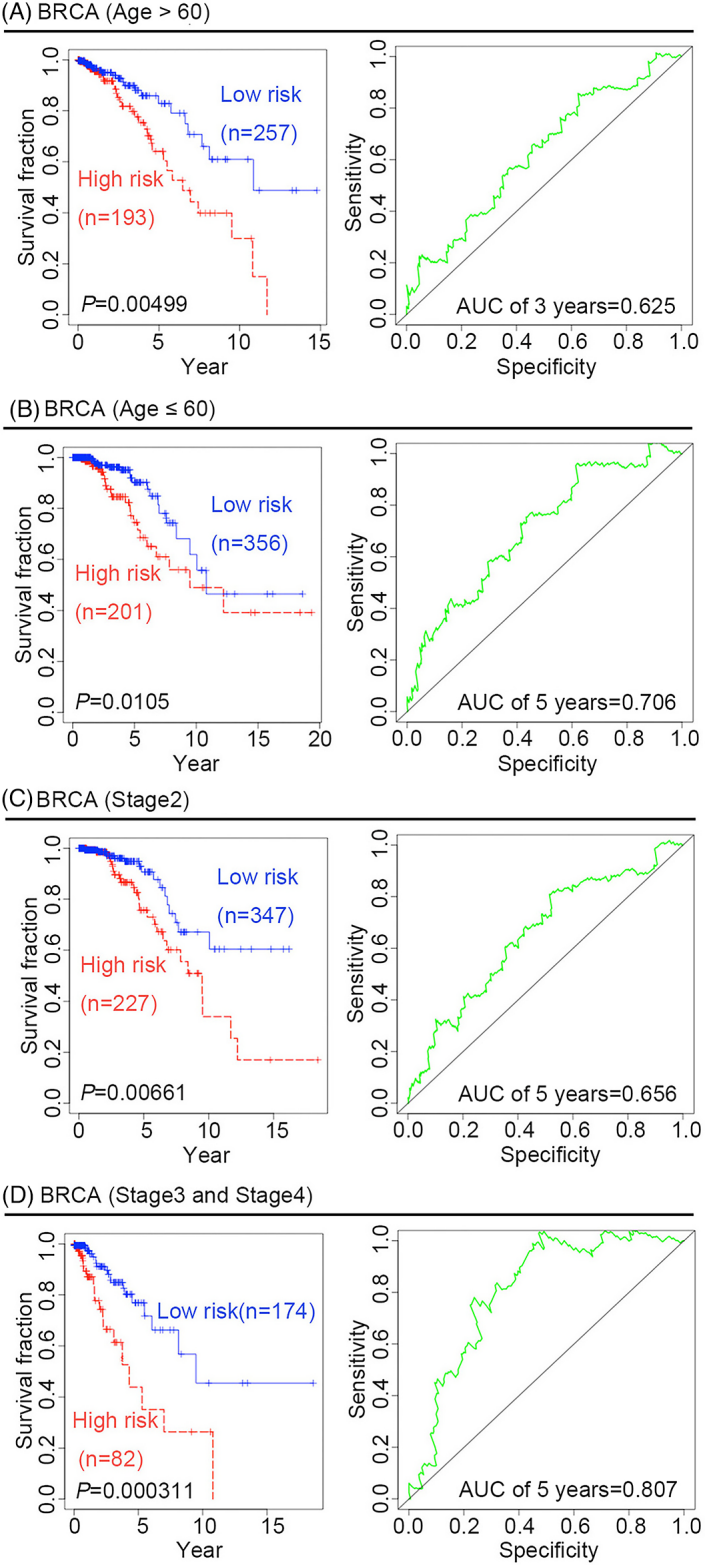
Overall survival analysis and ROC curves for BRCA patients at different ages and stage events. (A and B) OS analysis and ROC curves for older (age >60) and younger (age ≤60) BRCA patients. (C and D) OS analysis and ROC curves for stage 2 and stage 3/4 BRCA patients

To further confirm the predictive ability of the four‐gene signature, we performed survival analysis in different BRCA pathological subtypes. There are four pathological subtypes of human BRCA: estrogen receptor (ER)‐positive BRCA, progesterone receptor (PR)‐positive BRCA, human epidermal growth factor receptor 2 (HER2)‐positive BRCA, and TNBC. Compared with low‐risk patients with BRCA, high‐risk patients have poor outcomes, suggesting that the predictive capacity of the four‐gene signature is independent of the pathological subtype of BRCA, the time‐dependent ROC curves for the 3‐year OS of patients with ER‐positive, PR‐positive, HER2‐positive, and TNBC BRCA were 0.748, 0.803, 0.85, and 0.651, respectively (Figure 2A–D).Given that age and tumor stage may be unfavorable factors for survival outcomes, it is necessary to further examine the predictive value of the four‐gene signature to predict survival outcomes for cancer patients in different age groups or at different tumor stages. Two age groups of patients with BRCA were selected in this study: an older group (age >60 years) and a younger group (age ≤60 years). KM curves and log‐rank test suggested that the four‐gene signature might be more suitable for predicting survival outcomes in the older group than in the younger group. In fact, the ROC for the 3‐year OS in the older group was 0.625, whereas the ROC for the 5‐year OS in the younger group was 0.706 (Figure 3A,B). Considering the different stages of BRCA from the American Joint Committee on Cancer (AJCC), the four‐gene signature was used to predict the survival outcomes of patients with cancer at different stages, namely stages 1, 2, 3, and 4. KM curves and log‐rank tests indicated that the four‐gene signature might be more robust for patients with BRCA at stages 2, 3, and 4 than for those at stage 1. The ROC for the 5‐year OS of stage 2 patients was 0.656, while that for the 5‐year OS of stage 3 and 4 patients was 0.807 (Figure 3C,D). Both univariate and multivariate Cox regression analyses were performed to assess whether the four‐gene prognostic signature could serve as an independent prognostic factor. In addition to the risk score, covariates included clinical risk factors, such as age and tumor stage. Univariate Cox regression analysis showed that the risk score (hazard ratio [HR]: 1.955; 95% confidence interval [CI]: 1.467–2.605; *p* = 0.00000476), age (HR: 1.031; 95% CI: 1.015–1.048; *p* = 0:000148), and tumor stage (HR: 1.834; 95% CI: 1.410–2.384; *p* = 0.00000605) were significantly associated with OS in patients with BRCA. Multivariate Cox regression analysis confirmed that the risk score (HR: 1.893; 95% CI: 1.393–2.574; *p* = 0.0000463) was independent of age (HR: 1.03; 95% CI: 1.013–1.046; *p* = 0.000385) and tumor stage (HR: 1.788; 95% CI: 1.386–2.307; *p* = 0.00000767) (Table 1).

**TABLE 1:** Univariable and multivariable Cox regression analyses for BRCA

Variables	Univariable model			Multivariable model		
	HR	95% CI	*p* Value	HR	95% CI	*p* Value
Risk score	1.955	1.467–2.605	4.76E‐06	1.893	1.393–2.574	4.63E‐05
Age	1.031	1.015–1.048	1.48E‐04	1.030	1.013–1.046	3.85E‐04
Stage event	1.834	1.410–2.384	6.05E‐06	1.788	1.386–2.307	7.67E‐06

Abbreviations: CI, confifidence interval; HR, hazard ratio.

### 3.2 Prognostic prediction for other cancers

**FIGURE 4:**
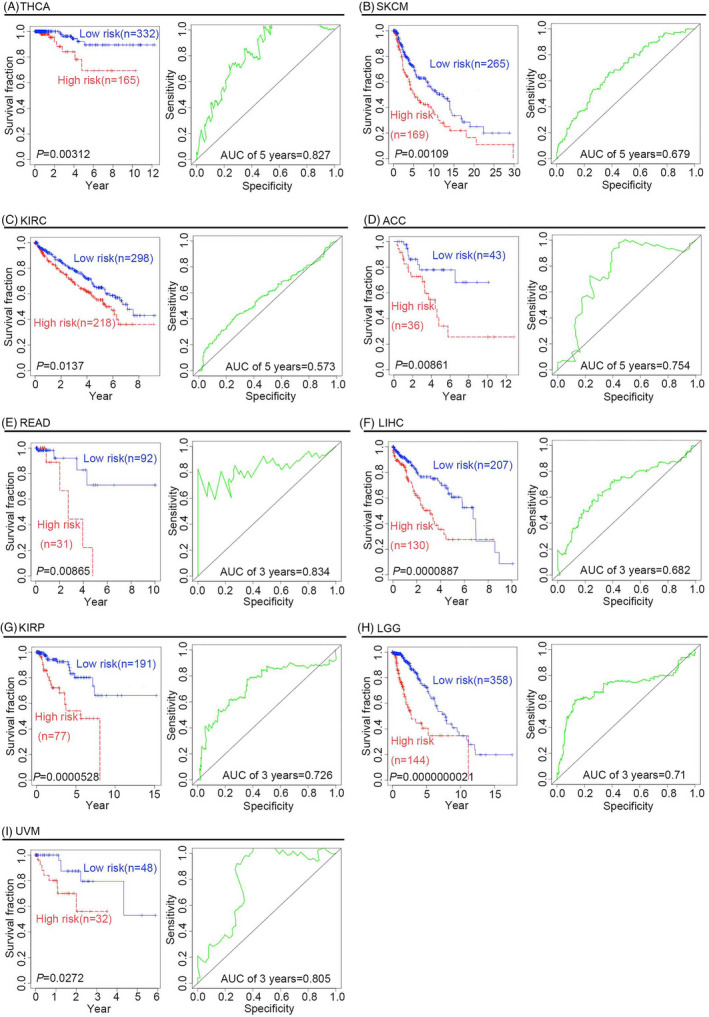
Overall survival analysis and ROC curves for patients with various cancers. The KM curves of the four‐gene signature (*CD8A*, *CD8B*, *TCF7*, and *LEF1*) showed that the risk score could be effectively used to divide patients into high‐ and low‐risk groups with distinct outcomes. The AUC of the ROC curve was used to evaluate the ability of the four‐gene signature to predict prognosis in terms of OS. (A) Patients with THCA. (B) Patients with SKCM. (C) Patients with KIRC. (D) Patients with ACC. (E) Patients with READ. (F) Patients with LIHC. (G) Patients with KIRP. (H) Patients with LGG. (I) Patients with UVM

To determine whether the four‐gene signature could predict the survival outcome of other tumors, Cox proportional hazard regression analysis was performed for nine types of cancers, namely THCA, LIHC, SKCM, LGG, KIRP, READ, ACC, KIRC, and UVM. As shown in (Figure 4A–I), both KM curves and log‐rank tests showed that the four‐gene signature was significantly associated with improved clinical outcomes of these cancers, and might be thus used as a powerful prognostic biomarker to predict survival outcomes of patients with these cancer types. The AUCs for the 5‐year OS of patients with THCA, SKCM, KIRC, and ACC were 0.827, 0.679, 0.573, and 0.754, respectively. The AUCs for the 3‐year OS of patients with READ, LIHC, KIRP, UVM, and LGG were 0.834, 0.682, 0.726, 0.805, and 0.71, respectively. Additionally, there were significant differences in the RFS outcomes of patients with THCA, LIHC, SKCM, LGG, KIRP, ACC, KIRC, and UVM, but not in those of patients with READ (*p* = 0.053) (Table 2). Furthermore, we investigated whether the four‐gene signature could serve as an independent prognostic factor for the outcome of other cancers, namely THCA, LIHC, SKCM, LGG, KIRP, READ, and KIRC. Covariates besides the risk score included clinical risk factors, such as sex, age, and AJCC tumor grade. Patients were divided into older (age >60 years) and younger (age ≤60 years) groups. Cancer stage events included stages 1, 2, 3, and 4. For LGG, LIHC, and SKCM patients, KM survival curves, log‐rank tests, univariable Cox regression analysis, and multivariate Cox regression analysis showed that the four‐gene signature has predictive value for different ages, stage events, or sexes, and thus could serve as an independent prognostic factor in these patients (Table 3 and Table 4; Figure S1, Figure S2, Figure S3 and Figure S4). However, the four‐gene signature exhibited poor predictive value for different ages, stage events, and sexes in patients with KIRC, KIRP, THCA, and READ, and thus could not serve as an independent prognostic factor for these cancer types (Table 5 and Table 6). Owing to the small sample size of patients with ACC or UVM, neither univariate nor multivariate Cox regression analyses were performed in this study.

**TABLE 2:** Log‐rank test analyses for RFS in cancers

Cancer types	*p* Value	ROC curves (AUC of 3 years)
ACC	4.03E‐03	0.618
BRCA	4.98E‐03	0.612
UVM	1.95E‐02	0.713
KIRC	1.81E‐04	0.590
KIRP	2.63E‐05	0.765
LGG	7.39E‐07	0.635
LIHC	1.95E‐03	0.650
SKCM	2.26E‐06	0.645
THCA	4.44E‐02	0.654
READ	5.53E‐02	ND

Abbreviation: ND, not detectable.

**TABLE 3:** Log‐rank test analyses for LGG, LIHC, and SKCM

Cancer type	Different groups	*p* Value (Log‐rank test)	ROC curves (AUC of 3 years)
LGG	Older group (age >60)	8.94E‐03	7.79E‐01^a^
	Younger group (age ≤60)	4.43E‐05	7.21E‐01
	Male	7.91E‐05	7.12E‐01
	Female	6.11E‐06	7.29E‐01
	Neoplasm histologic grade 2	1.88E‐02	5.69E‐01
	Neoplasm histologic grade 3	3.22E‐05	7.27E‐01
	RFS	7.39E‐07	6.35E‐01
LIHC	Older group (age >60)	2.35E‐02	5.89E‐01
	Younger group (age ≤60)	2.64E‐04	7.72E‐01
	Male	4.18E‐05	7.17E‐01
	Female	1.80E‐03	6.67E‐01
	Stage event 1	1.39E‐02	6.72E‐01
	Stage event 2	1.78E‐03	7.64E‐01
	Stage event 3, 4	2.99E‐02	6.77E‐01
	RFS	1.95E‐03	6.50E‐01
SKCM	Older group (age >60)	7.87E‐03	6.56E‐01
	Younger group (age ≤60)	4.25E‐02	7.35E‐01
	Male	8.41E‐04	6.80E‐01
	Female	2.77E‐02	6.70E‐01
	Stage event 1, 2	1.74E‐03	6.88E‐01
	Stage event 3, 4	5.48E‐03	6.92E‐01
	RFS	2.26E‐06	6.45E‐01

^a^ AUC of 2 years.

**TABLE 4:** Univariable and multivariable Cox regression analyses for LGG, LIHC, and SKCM

Cancer type	Variables	Univariable model			Multivariable model		
		HR	95% CI	*p* Value	HR	95% CI	*p* Value
LGG	Risk score	1.562	1.382–1.766	1.05E‐12	1.424	1.225–1.656	4.37E‐06
	Sex	1.127	0.735–1.726	5.84E‐01	0.922	0.590–1.442	7.23E‐01
	Age	1.070	1.052–1.089	2.65E‐14	1.068	1.048–1.088	4.34E‐12
	Neoplasm histologic grade	3.624	2.241–5.862	1.53E‐07	2.400	1.426–4.039	9.75E‐04
LIHC	Risk score	2.131	1.493–3.042	3.07E‐05	2.118	1.467–3.056	6.12E‐05
	Sex	1.507	0.928–2.446	9.74E‐02	1.241	0.747–2.060	4.04E‐01
	Age	1.028	1.007–1.050	9.17E‐03	1.024	1.003–1.045	2.47E‐02
	Stage event	1.217	0.926–1.598	1.59E‐01	1.151	0.877–1.511	3.11E‐01
SKCM	Risk score	2.401	1.699–3.394	7.02E‐07	2.387	1.683–3.385	1.06E‐06
	Sex	1.033	0.708–1.507	8.65E‐01	1.044	0.714–1.527	8.24E‐01
	Age	1.025	1.012–1.038	9.26E‐05	1.022	1.009–1.035	9.71E‐04
	Stage event	1.381	1.122–1.700	2.33E‐03	1.417	1.136–1.768	2.00E‐03

Abbreviations: CI, confifidence interval; HR, hazard ratio.

**TABLE 5:** Univariable and multivariable Cox regression analyses for KIRC, KIRP, THCA, and READ

Cancer type	Variables	Univariable model			Multivariable model		
		HR	95% CI	*p* Value	HR	95% CI	*p* Value
KIRC	Risk score	2.565	1.512–4.352	4.79E‐04	1.600	0.954–2.683	7.49E‐02
	Sex	1.075	0.762–1.516	6.82E‐01	1.118	0.789–1.586	5.31E‐01
	Age	1.032	1.017–1.047	1.64E‐05	1.037	1.021–1.054	7.26E‐06
	Stage event	1.955	1.689–2.263	2.56E‐19	1.934	1.660–2.252	2.31E‐17
KIRP	Risk score	1.619	1.332–1.968	1.33E‐06	1.849	1.406–2.431	1.09E‐05
	Sex	1.302	0.546–3.103	5.52E‐01	0.897	0.364–2.210	8.13E‐01
	Age	0.996	0.960–1.034	8.42E‐01	1.016	0.979–1.055	4.02E‐01
	Stage event	2.505	1.740–3.607	7.76E‐07	2.583	1.748–3.815	1.87E‐06
THCA	Risk score	1.460	1.181–1.806	4.76E‐04	1.091	0.831–1.432	5.32E‐01
	Sex	0.281	0.070–1.128	7.35E‐02	0.307	0.059–1.600	1.61E‐01
	Age	1.135	1.059–1.216	3.42E‐04	1.101	1.010–1.199	2.79E‐02
	Stage event	3.420	1.656–7.063	8.93E‐04	2.259	0.820–6.227	1.15E‐01
READ	Risk score	1.166	0.915–1.487	2.15E‐01	1.230	0.859–1.761	2.58E‐01
	Age	1.009	0.893–1.139	8.87E‐01	1.046	0.901–1.214	5.57E‐01
	Stage event	1.963	0.442–8.710	3.75E‐01	1.855	0.453–7.602	3.91E‐01

Abbreviations: CI, confifidence interval; HR, hazard ratio.

**TABLE 6:** Log‐rank test analyses for KIRC, KIRP, THCA, and READ

Cancer types	Different groups	*p* value (Log‐rank test)	ROC curves (AUC of 3 years)
KIRC	Older group (age >60)	2.11E‐03	5.84E‐01
	Younger group (age ≤ 0)	2.65E‐01	ND
	Male	3.70E‐02	5.97E‐01
	Female	9.95E‐02	ND
	Stage event 1, 2	4.54E‐01	ND
	Stage event 3, 4	4.23E‐02	5.63E‐01
	RFS	1.81E‐04	5.90E‐01
KIRP	Older group (age >60)	3.27E‐01	ND
	Younger group (age ≤60)	1.39E‐06	7.66E‐01
	Male	1.88E‐02	6.64E‐01
	Female	1.98E‐06	9.52E‐01
	Stage event 1, 2	1.68E‐01	ND
	Stage event 3, 4	6.84E‐03	7.15E‐01
	RFS	2.63E‐05	7.65E‐01
THCA	Older group (age >60)	3.01E‐01	ND
	Younger group (age ≤60)	6.82E‐07	9.88E‐01
	Male	1.06E‐01	ND
	Female	1.37E‐02	8.15E‐01
	Stage event 1, 2	4.08E‐01	ND
	Stage event 3, 4	5.11E‐03	8.64E‐01
	RFS	4.44E‐02	6.54E‐01
READ	Older group (age >60)	3.12E‐02	7.79E‐01
	Younger group (age ≤60)	1.00E+00	ND
	Male	ND	ND
	Female	ND	ND
	Stage event 1, 2	ND	ND
	Stage event 3, 4	ND	ND
	RFS	5.53E‐02	ND

Abbreviation: ND, not detectable.

### 3.3 Expression profile analysis for the four genes in different cancers

**FIGURE 5:**
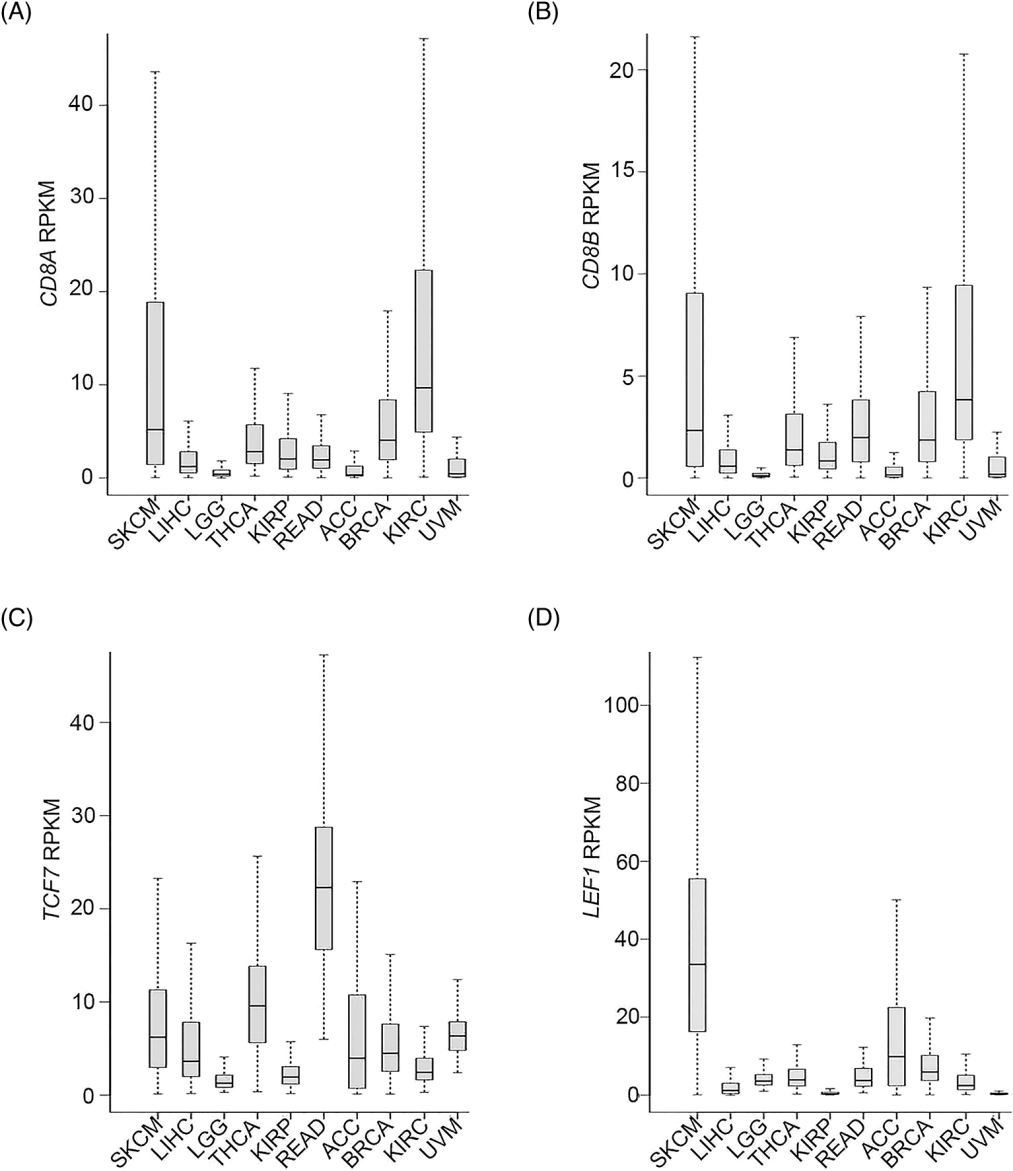
*CD8A*, *CD8B*, *TCF7*, and *LEF1* expression in RPKM in different cancer tissues. expression of the four signature genes in SKCM, LIHC, LGG, THCA, KIRP, READ, ACC, BRCA, KIRC, and UVM tissues. The bottom, middle, and top lines in each box correspond to the 25th, 50th, and 75th percentiles, respectively. (A) *CD8A*. (B) *CD8B*. (C) *TCF7*. (D) *LEF1*

We further examined the expression patterns of the four signature genes in the 10 types of cancers under investigation based on RPKM analysis. The 25th, 50th, and 75th percentiles are shown in (Figure 5A–D). Taking the median values as an example, *TCF7* was expressed in the range of 1.28–22.27 RPKM, *LEF1* in the range of 0.31–33.53 RPKM, *CD8A* in the range of 0.32–9.68 RPKM, and *CD8B* in the range of 0.10–3.84 RPKM. Therefore, the expression profiles of the four genes were consistent in the different cancers.

To detect the logarithm fold change (logFC) in the expression of the four genes *TCF7*, *LEF1*, *CD8A*, and *CD8B* in different cancers, we investigated the transcriptomic profiles retrieved from the TCGA database. Compared with that in normal tissues, *TCF7* expression in READ, KIRC, and LIHC tissues was higher, while being lower in BRCA tissues; in addition, *LEF1* was highly expressed in BRCA, READ, KIRC, and LIHC tissues, but was expressed at lower levels in KIRP tissues; finally, both *CD8A* and *CD8B* were expressed at higher levels in BRCA, KIRC, and KIRP tissues, while *CD8A* was expressed at lower levels in THCA and READ tissues (Table 7).

**TABLE 7:** Expression foldchanges in different cancers

Gene	Cancer	logFC	*p*‐Value	FDR
*TCF7*	BRCA	−0.736	9.52E‐13	2.25E‐12
	KIRP	0.356	9.47E‐02	1.27E‐01
	READ	2.227	6.74E‐17	1.69E‐15
	LIHC	0.945	9.39E‐06	2.28E‐05
	KIRC	0.976	1.08E‐11	2.62E‐11
	THCA	0.091	5.06E‐01	5.56E‐01
*LEF1*	BRCA	2.033	4.69E‐48	4.31E‐47
	KIRP	−1.266	1.02E‐06	3.13E‐06
	READ	2.110	7.66E‐07	4.42E‐06
	LIHC	3.012	2.30E‐21	3.89E‐20
	KIRC	1.157	4.15E‐09	8.60E‐09
	THCA	0.287	5.75E‐02	7.71E‐02
*CD8A*	BRCA	0.620	2.45E‐05	3.81E‐05
	KIRP	1.756	2.42E‐06	7.02E‐06
	READ	−0.820	2.83E‐02	5.20E‐02
	LIHC	−0.208	4.02E‐01	4.53E‐01
	KIRC	3.636	1.16E‐46	1.49E‐45
	THCA	−0.950	5.48E‐08	1.63E‐07
*CD8B*	BRCA	0.654	6.57E‐05	9.91E‐05
	KIRP	2.015	2.71E‐06	7.80E‐06
	READ	0.051	8.98E‐01	9.25E‐01
	LIHC	0.214	4.48E‐01	4.98E‐01
	KIRC	3.446	7.15E‐41	7.05E‐40
	THCA	−0.435	6.55E‐02	8.70E‐02

### 3.4 GO and KEGG pathway enrichment analyses

**FIGURE 6:**
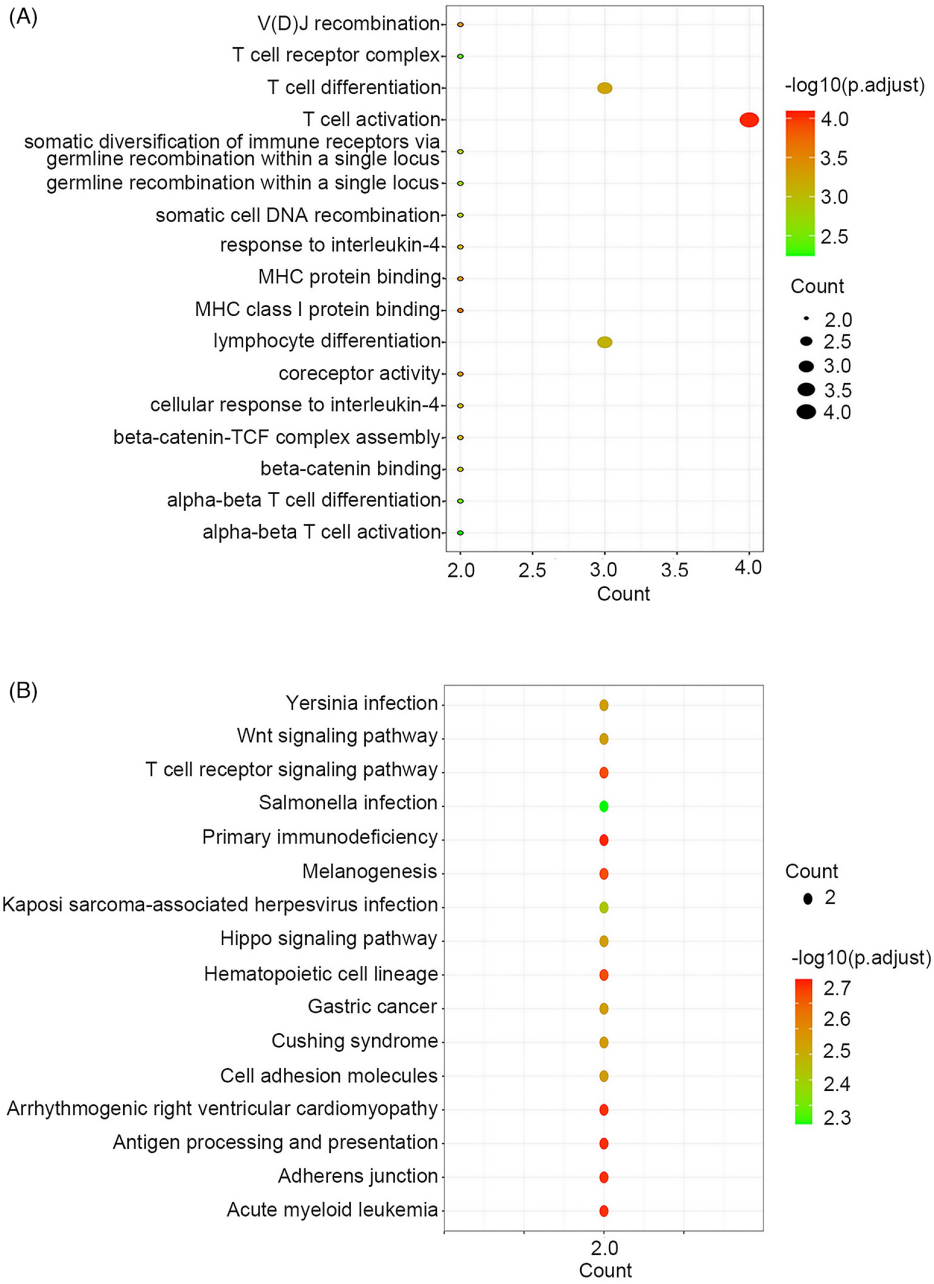
Gene ontology and KEGG pathway enrichment analyses. (A) GO enrichment analysis for *CD8A*, *CD8B*, *TCF7*, and *LEF1*. (B) KEGG pathway analysis for *CD8A*, *CD8B*, *TCF7*, and *LEF1*

To confirm the functions of *TCF7*, *LEF1*, *CD8A*, and *CD8B*, GO and KEGG enrichment analyses were performed. GO enrichment analysis showed that *CD8A*, *CD8B*, *TCF7*, and *LEF1* are involved in T‐cell activation, T‐cell differentiation, lymphocyte differentiation, V(D) J recombination, MHC class I protein binding, MHC protein binding, and coreceptor activity (Figure 6A). KEGG enrichment analysis further indicated that these four genes were mainly associated with the following KEGG terms: T‐cell receptor signaling pathway, primary immunodeficiency, melanogenesis, hematopoietic cell lineage, arrhythmogenic right ventricular cardiomyopathy, adherent junction, acute myeloid leukemia, antigen processing, and presentation (Figure 6B).

### 3.5 The four‐gene signature as an immune‐associated prognostic signature for cancers

**FIGURE 7:**
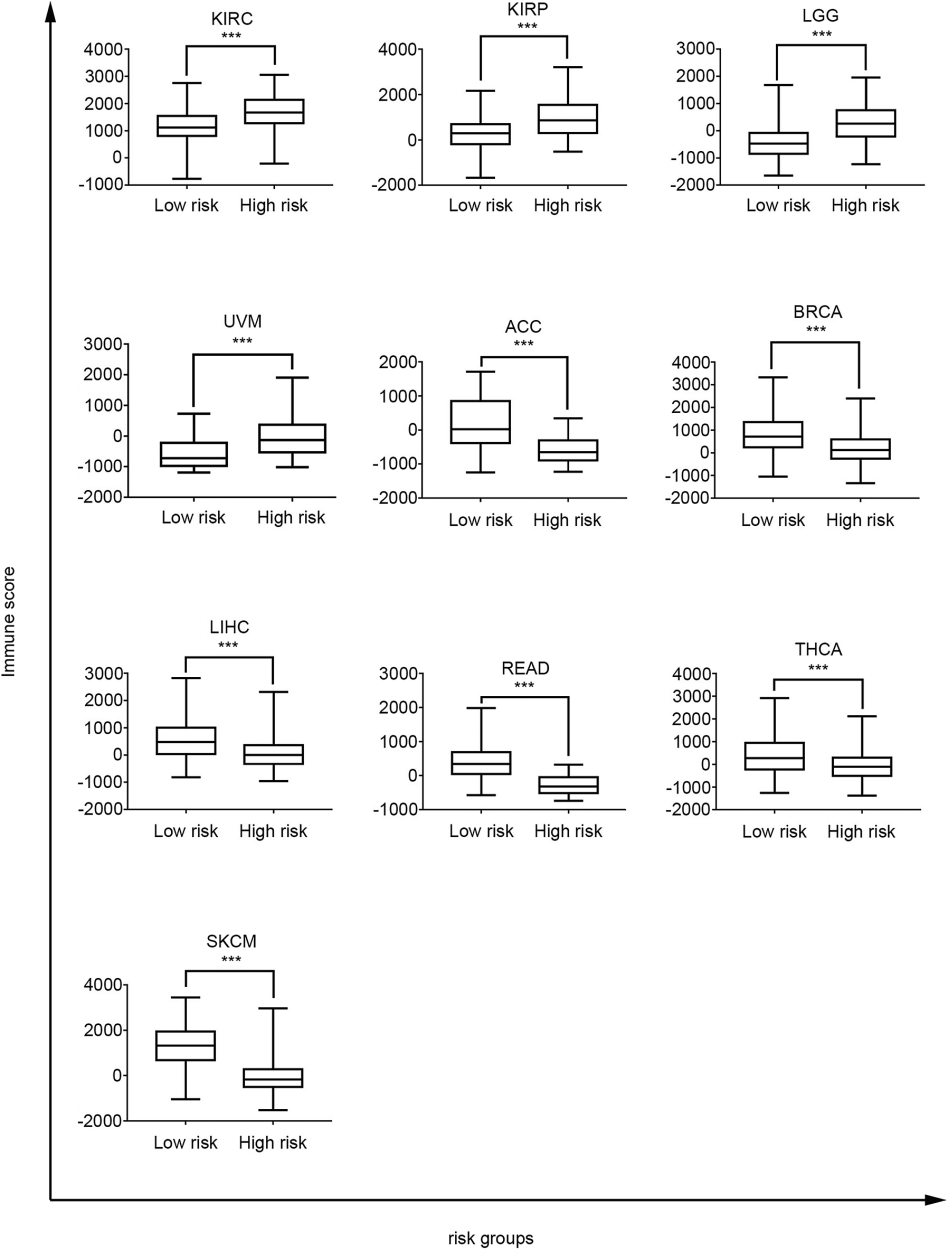
Immune Scores of High‐ and Low‐Risk Groups in Different Cancers. The four‐gene signature (*CD8A*, *CD8B*, *TCF7*, and *LEF1*) was associated with the immune score in KIRC, KIRP, LGG, UVM, ACC, BRCA, LIHC, READ, THCA, and SKCM (two‐tailed *t* test, *p* < 0.001)

**FIGURE 8:**
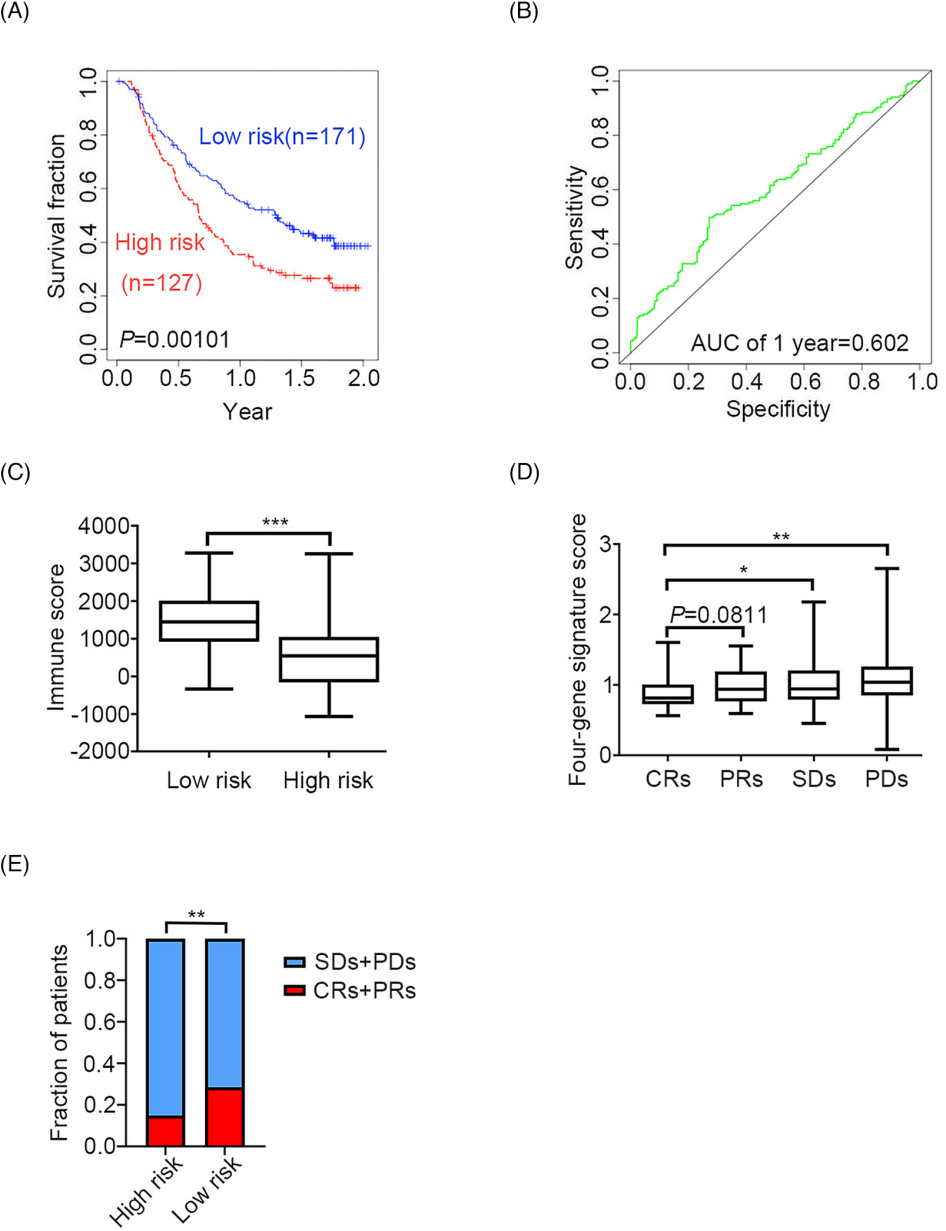
Validation of the Four‐Gene Signature (*CD8A*, *CD8B*, *TCF7*, and *LEF1*) in mUC. (A and B) OS analysis of the four‐gene signature (*CD8A, CD8B, TCF7, and LEF1*) in mUC and ROC curves for the 1‐year OS. The KM curves and log‐rank tests showed that the four‐gene signature is positively associated with the survival outcome of mUC patients. (C) The four‐gene signature (*CD8A, CD8B, TCF7, and LEF1*) is associated with immune score in mUC patients (two‐tailed *t* test, ***p < 0.001). (D) The four‐gene signature (*CD8A, CD8B, TCF7, and LEF1*) score is positively associated with responses to ICB treatment, and such association is driven by the complete response subgroup (two‐tailed *t* test, *p < 0.05, **p < 0.01). (E) The four‐gene signature (*CD8A, CD8B, TCF7, and LEF1*) score is positively associated with response to atezolizumab (two‐sided Chi‐square test, **p < 0.01). CRs, complete response subgroup; PDs, progressive disease subgroup; PRs, partial response subgroup; SDs, stable disease subgroup

To investigate the immune‐related risk stratification of the four‐gene signature, the immune score, which represents the immune infiltration level, was obtained using the R package estimate. When comparing high‐ to low‐risk groups of cancer patients, the four‐gene signature was found to be significantly associated with the immune infiltration level in patients with 10 types of cancer (Figure 7). For example, in patients with mUC, the KM survival curves and log‐rank test revealed significant differences in survival outcomes (Figure 8A, B). Moreover, immune infiltration levels differed significantly between the high‐ and low‐risk groups in patients with mUC (*p* < 0.0001. Figure 8C). The four‐gene signature score was lower in the complete response subgroups (CRs) than in the stable disease subgroups (SDs, *p* = 0.0475) or partial disease subgroups (PDs, *p* = 0.0017) (Figure 8D). The four‐gene signature was positively associated with complete response to atezolizumab treatment, an anti‐PD‐L1 immunotherapy (Figure 8E. *p* = 0.0054). Collectively, our results suggest that the four‐gene signature can be used to predict the efficacy of immunotherapy and identify patients that could benefit from immunotherapy.

## 4 DISCUSSION

In many cancers, the presence of tumor‐infiltrating CD8^+^ T lymphocytes can be used to predict patient survival and response to immunotherapy.^24^, ^25^, ^26^, ^27^, ^28^, ^29^, ^30^, ^31^ The presence of tumor‐infiltrating lymphocytes, particularly CD8^+^ T cells, is a positive prognostic marker in multiple solid tumors, but these cells fail to effectively eliminate cancer cells. This is because not all CD8^+^ T‐cell subsets expand following ICB.^32^ Stem‐like CD8^+^ T cells, a subpopulation of CD8^+^ T cells that can express effector molecules, play critical roles in maintaining CD8^+^ T‐cell responses in human cancers.^47^ In fact, the proliferative burst derives almost exclusively from these “stem‐like” CD8^+^ T cells after ICB. Moreover, melanoma patients with a higher percentage of progenitor exhausted cells benefit from a more durable response to ICB therapy.^34^, ^36^, ^37^, ^38^, ^39^ In the current study, based on the transcriptomic profiles from the TCGA database, a multivariable Cox regression analysis was conducted to establish a novel four‐gene signature (*CD8A*, *CD8B*, *TCF7*, and *LEF1*), which serves as a biomarker of stem‐like CD8^+^ T cells and predicts the immune responses and clinical outcomes of patients with metastatic cancer prior to treatment.

First, KM survival curves and log‐rank tests were used to examine the predictive capacity of the four‐gene signature for patients with BRCA. The results revealed significant differences in both clinical survival outcomes and RFS outcomes between high‐risk and low‐risk patients, indicating that the four‐gene signature has a robust predictive capacity for breast cancers. The cross‐tumor predictive value of the four‐gene signature was assessed in other nine cancers, namely THCA, LIHC, SKCM, LGG, KIRP, READ, ACC, KIRC, and UVM. Both the KM curves and log‐rank tests proved that the four‐gene signature has prognostic value for these cancers. Therefore, the four‐gene signature can be used to predict clinical immune responses in a wide variety of cancers.

Both univariate and multivariate Cox regression analyses were performed to determine whether the four‐gene prognostic signature could serve as an independent prognostic factor. The results demonstrated that the four‐gene signature has predictive value for different ages, stage events, or sexes, which could serve as independent prognostic factors for patients with BRCA, LGG, LIHC, or SKCM. However, the four‐gene signature could not serve as an independent prognostic factor in patients with KIRC, KIRP, THCA, or READ in the current study. Regarding KIRC, KIRP, THCA, and READ, previous studies have shown that age and sex are closely associated with OS.^48^, ^49^, ^50^, ^51^ Except for READ patients, the four‐gene signature was significantly associated with improved RFS for patients with all considered cancer types. Additionally, neither univariate Cox regression analysis nor multivariate Cox regression analysis was performed in the current study for patients with ACC and UVM, owing to their small sample size.

Interestingly, *CD8A*, *CD8B*, *TCF7*, and *LEF1* cannot serve as therapeutic targets for these cancers because of their different expression profiles. GO enrichment analysis showed that *CD8A*, *CD8B*, *TCF7*, and *LEF1* are involved in T‐cell activation, T‐cell differentiation, lymphocyte differentiation, V(D) J recombination, MHC class I protein binding, MHC protein binding, and coreceptor activation. Furthermore, KEGG enrichment analysis indicated that the four genes were mainly associated with the following KEGG terms: T‐cell receptor signaling pathway, primary immunodeficiency, melanogenesis, hematopoietic cell lineage, arrhythmogenic right ventricular cardiomyopathy, adherent junction, acute myeloid leukemia, antigen processing, and presentation. In fact, CD8A and CD8B might be involved in directing the cell fate of immature double‐positive (CD4^+^CD8^+^) thymocytes towards two subsets of T cells: MHC class II‐restricted CD4^+^ helper T cells and MHC class I‐restricted CD8^+^ cytotoxic T cells.^52^ TCF7 and LEF1 are HMG transcription factors required for the early stages of thymocyte maturation.^53^, ^54^, ^55^, ^56^ Importantly, TCF7 and LEF1 play critical roles in the establishment of CD8^+^ T‐cell identity, as they are directly involved in genetic and epigenetic regulation resulting in an appropriate gene expression pattern for CD8^+^ T cells. Such regulatory functions mainly depend on their intrinsic histone deacetylase activity.^43^ Therefore, both TCF7 and LEF1 have versatile functions in regulating T‐cell development and differentiation, and are involved in CD8^+^ T‐cell maturation.^57^, ^58^

We further investigated whether the immune‐related risk stratification of the four‐gene signature correlated with the immune infiltration level in patients with 10 types of cancer, namely THCA, LIHC, SKCM, LGG, KIRP, READ, ACC, KIRC, BRCA, and mUC. The results revealed significant differences in immune infiltration levels between the high‐ and low‐risk groups of patients. Based on the efficacy and safety data of atezolizumab in mUC patients from the phase II IMvigor210 study, KM survival curve analysis and log‐rank tests were performed; the results demonstrated that the four‐gene signature had prognostic value for survival outcomes in mUC patients. Compared with those of the CRs groups, there were significant differences in the four‐gene signature risk scores of the SDs and PDs groups. These results demonstrated that the four‐gene signature could serve as a prognostic immunotherapy biomarker to predict the immune response in patients with cancer.

In summary, the novel four‐gene signature (*CD8A*, *CD8B*, *TCF7*, and *LEF1*) could serve as a predictive biomarker of the immune responses to ICB and clinical outcomes of patients with different cancers, including BRCA, THCA, LIHC, SKCM, LGG, KIRP, READ, ACC, KIRC, and UVM. The four‐gene signature could be widely used to optimize biomarkers of ICB responses, guide ICB therapy, and identify new immunotherapy targets for restoring immune responses. However, in the current study, the four‐gene signature was established based only on transcriptome analysis, and its combination with other biomarkers may yield a more promising tool for the prediction of immune responses to checkpoint blockades in multiple cancers in the future.

## AUTHOR CONTRIBUTIONS

(I) Conception and design: HZ, SZ, and YL; (II) Administrative support: HZ and SZ; (III) Provision of study materials or patients: YL, MN, and LL; (IV) Collection and assembly of data: MN, LL, JW, and ZT; (V) Data analysis and interpretation: YL, MN, SZ, and HZ; (VI) Manuscript writing: All authors; (VII) Final approval of manuscript: All authors.

## CONFLICT OF INTEREST

The authors declare that they have no competing interest.

## Supporting information


Figure S1

Figure S2

Figure S3

Figure S4

Table S1

## Data Availability

The datasets used and/or analyzed during the current study are available from the corresponding author upon reasonable request. Haisheng Zhou https://orcid.org/0000‐0002‐4218‐8641.
