# Supplementary figures and images for: A novel four‐gene signature predicts immunotherapy response of patients with different cancers
Source: J Clin Lab Anal. 2022 May 19;36(7):e24494. doi: 10.1002/jcla.24494 (PMC9279975; doi:10.1002/jcla.24494)

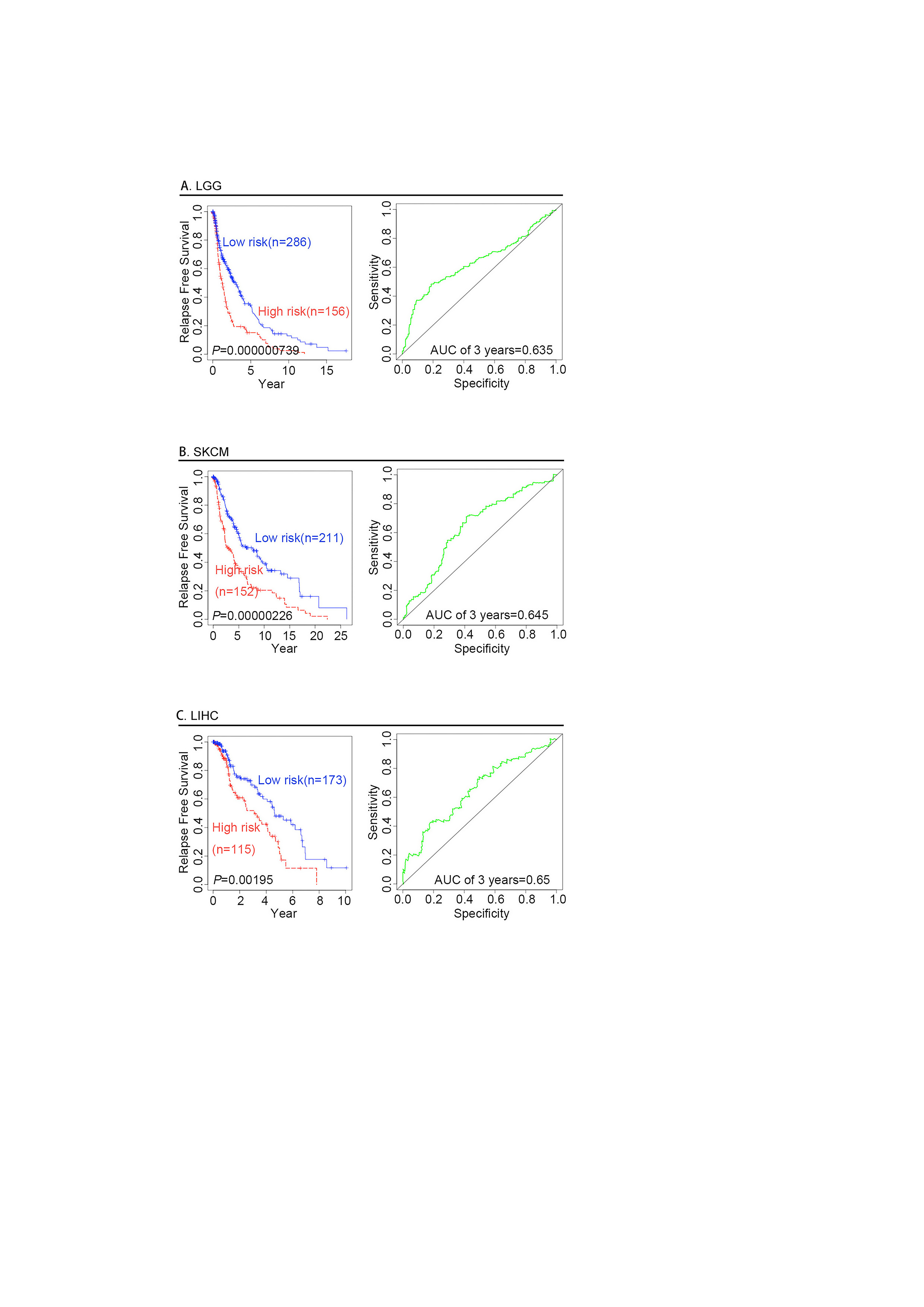

Supplement: Supplementary file 1 — Figure S1 [file JCLA-36-e24494-s002.tif]

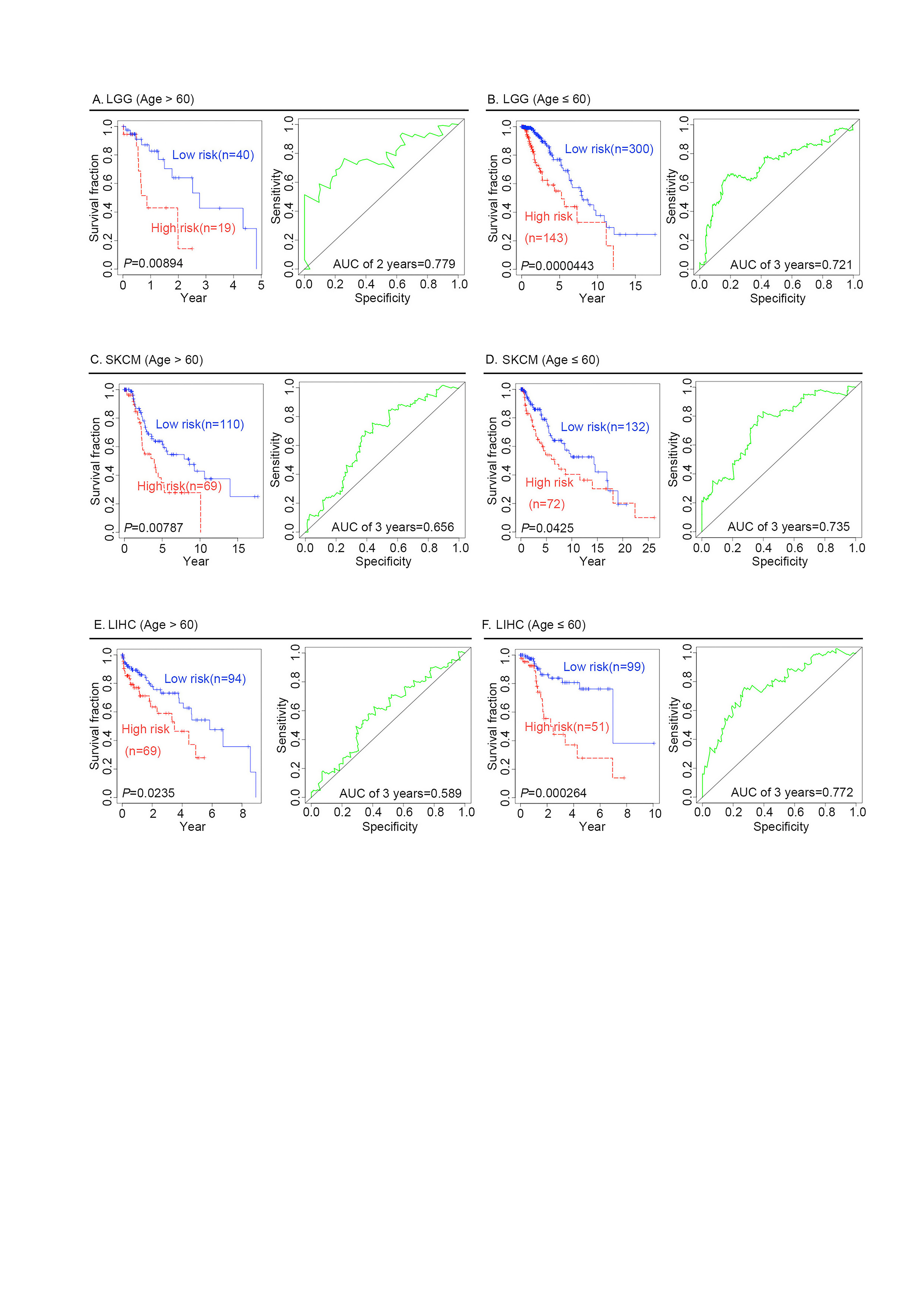

Supplement: Supplementary file 2 — Figure S2 [file JCLA-36-e24494-s001.tif]

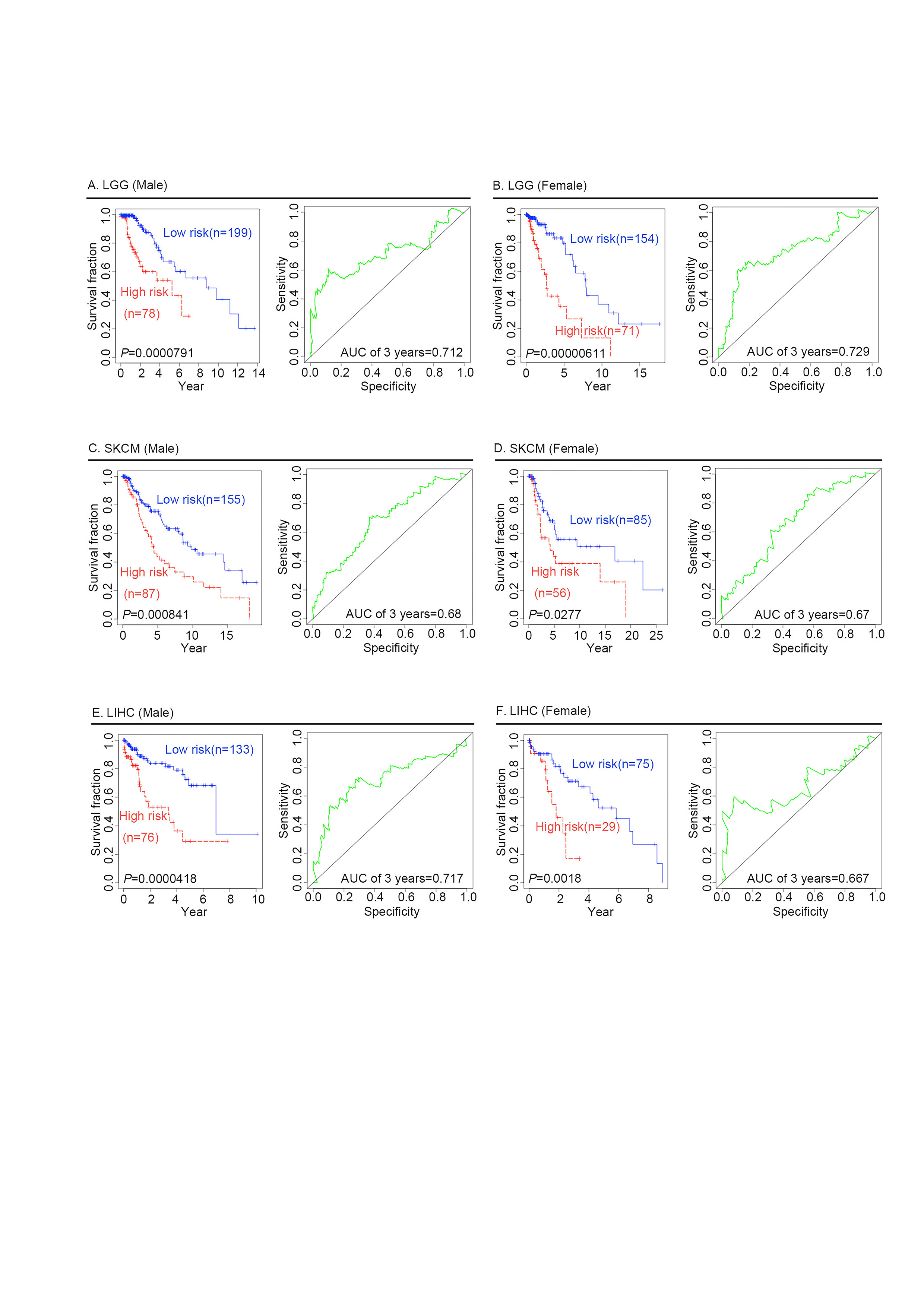

Supplement: Supplementary file 3 — Figure S3 [file JCLA-36-e24494-s004.tif]

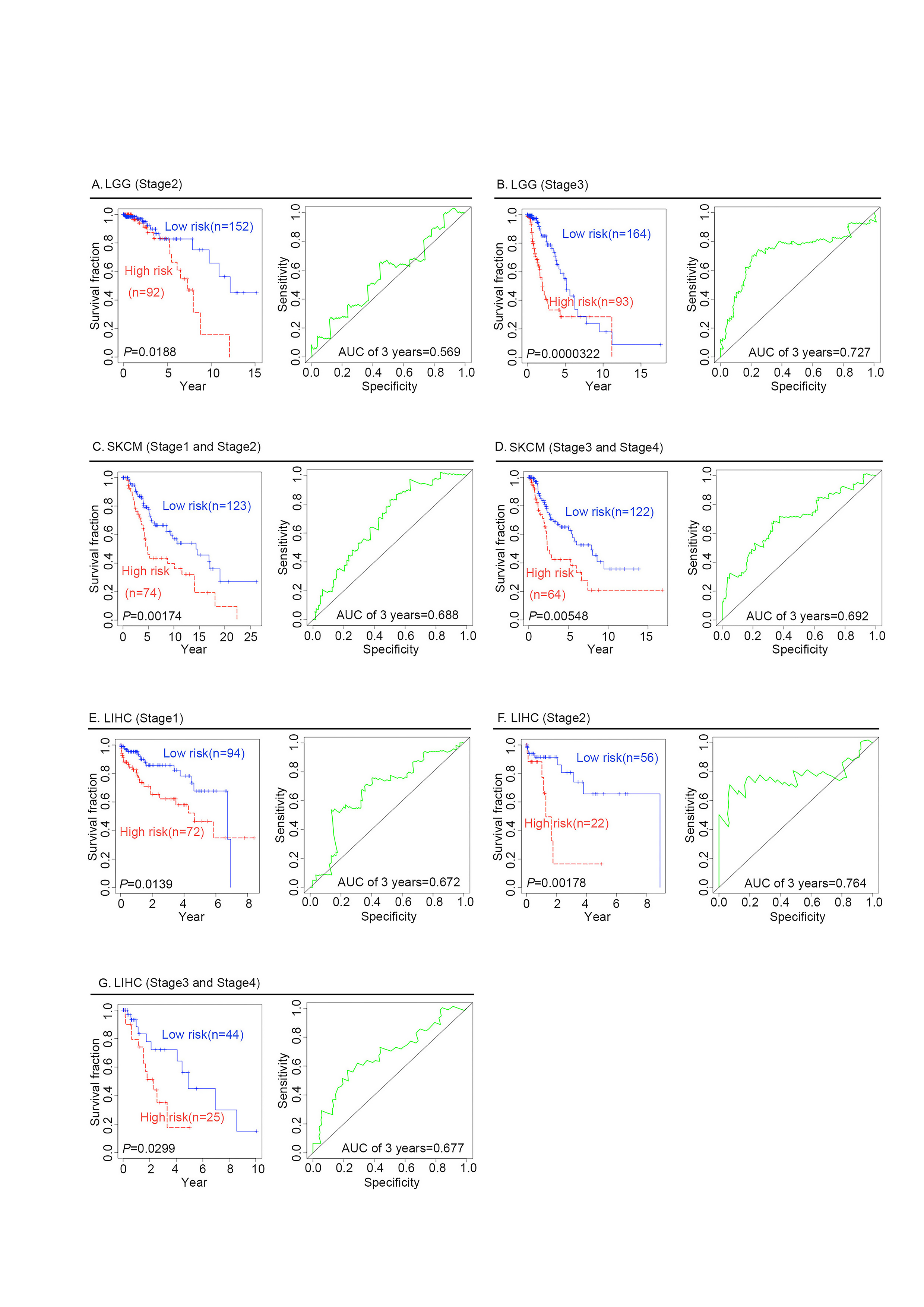

Supplement: Supplementary file 4 — Figure S4 [file JCLA-36-e24494-s005.tif]
