# Supplementary material for: A novel four‐gene signature predicts immunotherapy response of patients with different cancers
Source: J Clin Lab Anal. 2022 May 19;36(7):e24494. doi: 10.1002/jcla.24494 (PMC9279975; doi:10.1002/jcla.24494)
Supplement: Supplementary file 5 — Table S1 [file JCLA-36-e24494-s003.docx]

Table S1. Sample size and characteristics.

| Cancer type | Tumor sample size | Normal sample size |
| --- | --- | --- |
| BRCA | 1109 | 113 |
| SKCM | 470 | 0 |
| LGG | 529 | 0 |
| KIRP | 288 | 32 |
| READ | 166 | 10 |
| KIRC | 538 | 72 |
| THCA | 510 | 58 |
| LIHC | 374 | 50 |
| ACC | 79 | 0 |
| UVM | 80 | 0 |
